# Body Composition in Adult Patients with Thalassemia Major

**DOI:** 10.1155/2016/6218437

**Published:** 2016-11-10

**Authors:** Marianna Vlychou, Evangelos Alexiou, Paschalis Thriskos, Ioannis Fezoulidis, Katerina Vassiou

**Affiliations:** ^1^Department of Radiology, University Hospital of Larissa, Medical School of Thessaly, Larissa, Greece; ^2^Department of Radiology, General Hospital of Larissa, Greece

## Abstract

*Objective*. To assess body composition in adult male and female patients with thalassemia major by dual-energy X-ray absorptiometry (DXA) and to compare the findings with a group of healthy age-matched controls.* Methods*. Our study group included sixty-two patients (27 males, mean age 36 years, and 35 females, mean age 36.4 years) and fifteen age-matched healthy controls. All patients had an established diagnosis of thalassemia major and followed a regular blood transfusion scheme since childhood and chelation treatment. Fat, lean, and bone mineral density (BMD) were assessed with dual-energy X-ray absorptiometry. Ferritin levels and body mass index of all patients and controls were also recorded. Student* t-*test and Wilcoxon test were performed and statistical significance was set at *p* < 0.05.* Results*. BMD and whole body lean mass are lower in both male and female adult patients compared with controls (*p* < 0.01 in both groups), whereas whole body fat mass was found to have no statistically significant difference compared to controls. Regional trunk fat around the abdomen was found to be lower in male patients compared to controls (*p* = 0.02).* Conclusion*. Severe bone loss and diminished lean mass are expected in adult male and female patients with thalassemia major. Fat changes seem to affect mainly male patients.

## 1. Introduction

Thalassemia major, also known as Cooley's anemia, is a severe transfusion-dependent anemia and belongs to the group of beta-thalassemia autosomal recessive disorders. Beta-thalassemia is caused by the reduced (beta^+^) or absent (beta^0^) synthesis of the beta globin chains of the hemoglobin (Hb) tetramer, which is made up of two alpha globin and two beta globin chains (alpha_2_beta_2_) [1, 2]. Three clinical and hematological conditions of increasing severity are recognized and include beta-thalassemia carrier state, thalassemia intermedia, and thalassemia major. The beta-thalassemia carrier state is clinically asymptomatic. Apart from the rare dominant forms, subjects with thalassemia major are homozygotes or compound heterozygotes for beta^0^ or beta^+^ genes, subjects with thalassemia intermedia are mostly homozygotes or compound heterozygotes, and subjects with thalassemia minor are mostly heterozygotes. The incidence of the above disease is increased among Mediterranean countries and also in Middle East, Transcaucasus, Central Asia, Indian subcontinent, Far East, and Africa. There is a recognized protection of beta-thalassemia carriers against* Plasmodium falciparum* responsible for malaria, as it is indicated by its geographical distribution. However, the migration of populations has spread the gene worldwide [1, 2].

People suffering from thalassemia major require regular blood transfusions to survive and manage to reach quite prolonged life expectancy by virtue of modern therapy [2]. The major side effect of multiple transfusions is iron overload leading to secondary hemochromatosis that affects multiple organs such as heart, liver, and endocrine glands and is responsible for deficits in bone mineral acquisition [3–8]. Various studies have shown that children with thalassemia major suffer from growth retardation, impaired immune function, and low body mass index [8–10]. The above treatment-related complications disrupt the balance between osteoblasts and osteoclasts by interfering with various molecular mechanisms leading to osteoporosis and increased fracture risk during adulthood [11, 12]. Chelation treatment should be added routinely in order to prevent iron overload and its toxic effects [12, 13]. However, parenteral administration of chelate agents reduces patients' compliance and the longer survival rates lead to some degree of toxic systemic iron overload.

Body composition is influenced by many parameters including age, gender, endocrine system status, nutrition, and exercise [14]. It is well established that all the above parameters are impaired in patients with thalassemia major in addition to hyperactivity of bone marrow, iron overload, and diminished bone mineral density [7, 12]. However, there is limited published data regarding the body composition status in this population, especially during adulthood. Lean tissue mass has been shown to be highly correlated with bone mineral density until the middle age when fat mass begins to account for a larger variance thereafter [12]. DXA has been reported as a reliable modality to assess body composition in various patient groups, including thalassemia [15–18].

The purpose of the present study was to investigate body composition status in patients with thalassemia major during adulthood by the use of DXA and to correlate the findings with age-matched controls. We also sought to determine whether regional fat distribution at the trunk region around abdomen in patients with thalassemia major is comparable with those in controls and discuss the hypothesis that patients with thalassemia major may appear at risk for being underweight.

## 2. Materials and Methods

### 2.1. Study Populations

Sixty-two adult patients with the diagnosis of thalassemia major were referred to our laboratory from the Thalassemia Unit of our General Hospital for DXA assessment and formed our study group. The group included 27 males (mean age 36 years, age range 26–53 years) and 35 females (mean age 36.4 years, age range 21–47 years). All women had menstrual cycle. All patients had an established diagnosis of thalassemia major and followed a regular blood transfusion scheme since childhood, that is, systematic transfusion of two units of leukoreduced, packed red blood cells at 2- to 3-week intervals. All patients were instructed to undergo chelation treatment with variable compliance. Splenectomy was reported in 21/62 patients. The control group included 5 healthy age-matched males and 10 healthy age-matched females. The procedures related to the study were approved by the Institutional Review Board at the University Hospital. The participants were informed of the study requirements and informed consent was obtained prior to participation.

### 2.2. Body Composition and Bone Measurements

Body weight was measured to the nearest 0,1 Kg with patient in light clothing and shoes removed. Height was measured using a wall-mounted stadiometer and recorded to the nearest 0.1 cm. DXA examination was performed in a Hologic Discovery QDR Series Densitometer (Hologic Inc., Bedford, MA). The device was calibrated daily, according to the manufacturer's instructions for quality control with coefficients of variation of 0.2–0.5%. Body mass index (BMI) was calculated as kg/m^2^. Lumbar vertebrae L1–L4 and right hip were scanned and subsequently whole body DXA was performed to assess body composition, for example, body fat (kg, %) and lean mass (kg). Regional measurements were performed by the use of an arbitrary region of interest that was placed manually covering the abdomen and regional data regarding trunk fat around the abdomen (kg, %) and lean mass (kg) were calculated in both patients and controls. BMD and BMC were also calculated regarding the whole body and the subregion of the trunk around the abdomen. All patients and controls were placed at the standard positioning for whole body scanning with the subject in supine position, feet strapped together, per manufacturer's guidelines, and hands placed flat on the table adjacent to the side of the body. Average scanning time was approximately 7 min. To reduce variance in the data, DXA scans were performed and analyzed by a single operator.

Serum ferritin levels were determined in all patients and controls by blood samples. Body mass index was calculated for each subject manually.

### 2.3. Statistical Analysis

In this case-control study, parametric (Student* t*-test) and nonparametric (Wilcoxon) statistical tests were used to quantify the effects studied between patient and control groups. Height* z *score was calculated for patients and controls among men and women. Mean and standard deviation data are found in Benetou et al. [19]. Pearson correlation was estimated in order to quantify the dependence between lean body mass, bone mineral density* z* scores, height* z* scores, and serum ferritin. In addition to that whole body lean mass and regional lean mass at the abdominal area were correlated with calcitonin, osteocalcin, parathormone (PTH), IGF-1, and 25-OH vitamin D. Typically, correlation between two variables with values greater than 0.6 shows positive dependence, meaning that both variables increase or decrease together; values less than −0.6 indicate that if one variable increases the other decreases and values in between indicate independence. *p* values less than 0.05 were considered statistically significant. The software package R for statistical analysis was used to carry out all calculations.

## 3. Results

Table 1 shows demographic and clinical characteristics of patients with thalassemia major.  Table 2 shows the body composition variables of adult male and female patients with thalassemia major and controls.  Table 3 shows height and height *z* scores in patients and controls. Various correlations between ferritin, calcium hormones, lean body mass, and bone mineral density are shown in Tables 4, 5, and 6.

Ferritin levels were found to be abnormal by the use of nonparametric statistical Wilcoxon test in both male and female patients compared to controls. The statistical significance was estimated to be *p* = 0.028 in the male group and *p* < 0.0001 in the female group compared to controls, respectively.

**Figure 1:**
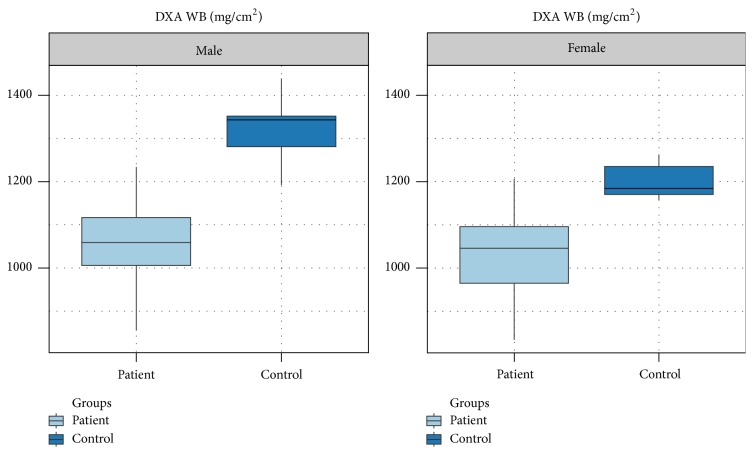
Bone mineral density of the whole body determined with DXA in male and female patients with thalassemia major compared with controls. There is established osteoporosis in both male and female patient groups, compared to controls (*p* = 0.001 in males, *p* < 0.0001 in females, resp.).

Body mass index (BMI) was lower only in the male patients, compared to controls (*p* = 0.015). The female patient group exhibited non-statistically significant difference compared to healthy age-matched controls. Bone mineral density (BMD) of the whole body was found to be severely diminished in both male and female patients compared to controls, with statistically significant difference (*p* = 0.001 for the male group and *p* < 0.0001 for the female group, resp.) (Figure 1).

**Figure 2:**
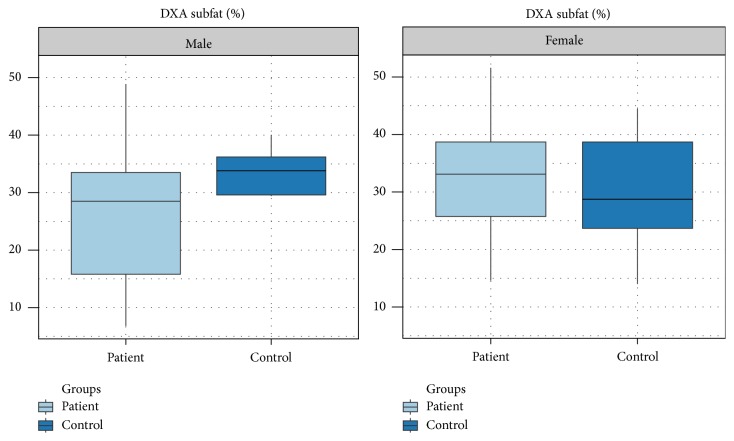
Percentage of regional trunk fat determined with DXA in male and female patients with thalassemia major compared with controls. There is a statistically significant difference in the male group (*p* = 0.02) but there is no difference in the female group of patients.

**Figure 3:**
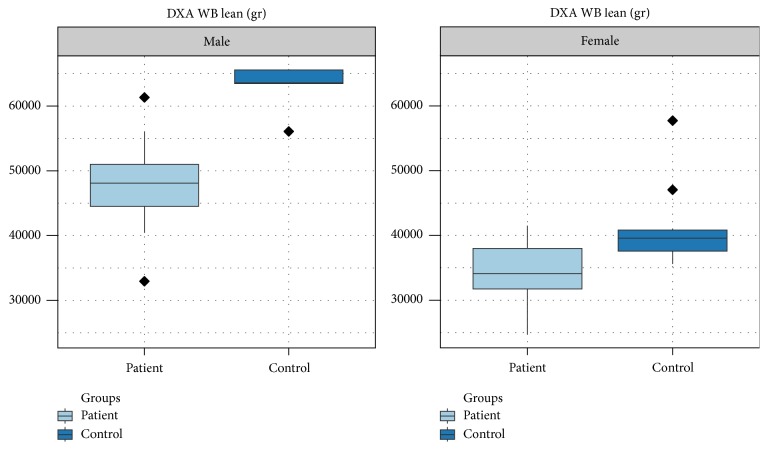
Lean mass of the whole body determined with DXA in male and female patients with thalassemia major compared with controls. Both male and female patients show a statistically significant reduction of the lean mass (*p* < 0.01 in both groups).

The overall fat, expressed in whole body fat percentage and in Kg, is lower in male patients with thalassemia major compared to controls but is not showing a statistical effect (*p* = 0.07). Likewise, the whole body fat in women shows no difference between patients and controls (*p* = 0.89). Regarding the regional fat around the abdomen that was expressed as trunk fat percentage, male patients were found to have statistically significantly less fat compared to controls (*p* = 0.02), whereas no difference was observed between female patients and controls. The whole body lean mass was found to be severely diminished in both male and female groups, compared to controls (*p* < 0.01 for males and females, resp.) (Figures 2 and 3).

## 4. Discussion

This study investigated body composition in a sample of adult male and female patients with thalassemia major by use of DXA and compared the findings with healthy age-matched controls. There is limited data in the literature exploring body composition status in such patients [20], although the fact of multifactorial osteoporosis mainly induced by iron overload and secondary endocrinopathies that interfere with molecular paths of bone metabolism is well established [21, 22]. The majority of our patients suffered from hypothyroidism and received thyroxine and more than half of the female patients were under estrogen therapy in order to maintain menstrual cycle. There is also a reported concept of underweight status of thalassemic patients, especially among childhood and adolescence, due to impaired nutrition and reduced physical activity [21].

The present assessment of body composition among adult patients with thalassemia major yielded some observations regarding fat and lean mass. At first, whole body fat percentage showed no statistically significant difference between patients and controls. These findings agree with previously reported data, which observed that transfused subjects with thalassemia had a higher percentage of body fat compared with nontransfused subjects after controlling for age, sex, and ethnicity [21]. However, male patients were found to have less body fat compared to controls which though not statistically significant showed a trend of diminished adipose tissue. There was one male patient with BMI = 30.4, consistent with obesity and one male patient with BMI = 16.1, consistent with being underweight. All other patients were found to be within healthy weight limits. Female patients were found to have similar percentage of overall fat compared to controls. There was one obese female patient with BMI = 33.2 and six female patients with BMI > 25, consistent with being overweight. This finding has not been previously reported, possibly because studies for patients with thalassemia major focus on younger age groups.

However, there are some studies that have explored the coexistence of metabolic syndrome in patients with thalassemia minor and it has been suggested that the prevalence of metabolic syndrome showed no relationship with sex and age and these patients had just higher BMI [23]. It has also been reported that patients with thalassemia minor have a better lipidemic and metabolic profile; however, women seem to be at least equally protected against cardiovascular risk compared with men [24]. Interestingly, the regional distribution of trunk fat around the abdomen shows a statistically significant difference between male patients and controls (*p* = 0.02), which is not observed among female patients and controls (*p* = 0.46). It may be postulated that sex can be an important factor but understanding the mechanisms of metabolic paths in patients with thalassemia major needs further to be explored.

The final stature between patients and controls was found to be significantly different (*p* = 0.04) between female patients and controls; namely, patients with thalassemia major reached a shorter height as adults (Table 3). However, this was not observed among male patients and controls (*p* = 0.94). The small sample of male controls may affect this observation that needs to be further investigated in larger groups.

Another observation is that lean mass is lower in both male and female adult patients compared with controls (*p* < 0.01 in both groups). Approximately two-thirds of our patients (41/62) have not performed splenectomy. Despite that, lean mass is a factor that seems to be impaired in adult patients of both sexes with thalassemia major. Our data also confirmed that the total BMD of patients is lower compared to controls. There are published data in the literature which prove that osteoporosis is a well-recognized side effect in thalassemic patients and explain the molecular paths that are implicated in this procedure [8, 11, 16, 25].

Correlation studies were performed between calcium hormones and lean body mass of thalassemic patients (Table 5), which showed no statistically significant correlation. Interestingly, there was a statistically significant correlation among male patients between abdominal lean mass and IGF-1 (*p* = 0.02) (Table 6).

Wong et al. [22] recently reported a correlation of hypogonadism with body composition in patients with thalassemia and found that this endocrine disorder attenuates the strength of the muscle-bone relationship in males but strengthens the positive correlation of skeletal muscle mass and fat mass in females. Hypogonadism, diabetes mellitus, hypothyroidism, and hypoparathyroidism are recognized complications among patients with thalassemia [5]. Improved chelation therapy has been reported to improve the rate of new endocrine disorders and stabilize preexisting disease [12, 13]. Patients with poor compliance regarding chelation therapy have an increased risk of developing endocrine disorders that affect body composition status. Correlation studies between lean body mass, bone mineral density* z* scores, and height* z* scores serum ferritin levels between male and female patients showed no statistical significance (Table 4).

There are some limitations in this study. First, the number of patients is relatively small because the referral Hospital Unit is responsible for a maximum of 100 patients with thalassemia major. Second, we were able to recruit only fifteen controls due to restricted availability of performing the above scans for research purposes. The lack of a digital archive for each patient also made it difficult to gather clinical information regarding endocrine status of all patients that formed our study group and correlate them with body composition findings.

Body composition in adult patients with thalassemia major seems to have distinct features compared to that among pediatric populations and adolescents who suffer from this disease. It may be suggested that a severe decrease of whole body bone mineral density and lean mass is a constant finding in both male and female patients whereas regional trunk fat around the abdomen is lower only in male patients compared to controls.

## Figures and Tables

**Table 1:** Characteristics of patients with thalassemia major.

	Male patients (*n* = 27)	Female patients (*n* = 35)
Height (cm)	173.3 (154–186)	160.4 (150–174)
Weight (kg)	69.7 (53–94)	59 (44–80)
BMI (m/kg^2^)	23.2 (16.1–30.4)	22.7 (17.2–33.2)
Age (years)	36 (26–53)	36.4 (21–47)
Ferritin levels (ng/mL)	1120.43 (103–3688)	1354.2 (80–4388)

Normal value range for ferritin levels.

Males: 12–300 ng/mL (nanograms per milliliter) and females: 12–150 ng/mL.

**Table 2:** *p* values of body composition variables between male and female patients and controls.

Variable	*p* values Male patients-controls (*n* = 27)	*p* values Female patients-controls (*n* = 35)
Ferritin levels (ng/mL)	**0.028**	**<0.0001**
BMD WB (mg/cm^2^)	**0.001**	**<0.0001**
WB (*T*-score)	**0.001**	**<0.0001**
WB fat (%)	0.07	0.87
WB fat (gr)	0.07	0.89
WB lean (gr)	**<0.01**	**<0.01**
Subtrunk fat (%)	**0.02**	0.46
BMD subtrunk (mg/cm^2^)	0.06	**<0.0001**
BMI (Kgr/m^2^)	**0.015**	0.38

*p* < 0.05 is statistically significant.

**Table 3:** Height and height *z* score.

Group	Gender	*N*	Height	
			Actual	*z* score
Patients	Male	27	172.37 ± 7.29	0.73 ± 1.00
	Female	35	160.48 ± 6.57	0.96 ± 1.06

Controls	Male	5	172.80 ± 11.18	0.79 ± 1.54
	Female	10	164.90 ± 5.30	1.69 ± 0.85

**Table 4:** Correlation between ferritin and lean body mass, bone mineral density *z* scores, and height *z* scores.

Group	Gender	Ferritin and lean body mass	Ferritin and BMD (*z* score)	Ferritin and height (*z* score)
Patients	Male	0.04 (*p* = 0.84)	0.13 (*p* = 0.49)	−0.10 (*p* = 0.59)
	Female	0.22 (*p* = 0.21)	0.02 (*p* = 0.93)	0.03 (*p* = 0.89)

Controls	Male	0.27 (*p* = 0.66)	0.51 (*p* = 0.38)	0.40 (*p* = 0.5)
	Female	0.17 (*p* = 0.64)	0.13 (*p* = 0.72)	0.34 (*p* = 0.32)

**Table 5:** Correlation between lean body mass and calcium hormones.

Group	Gender	Calcitonin	Osteocalcin	PTH	IGF-1	25-OH vitamin D
Patients	Male	0.02 (*p* = 0.92)	0.10 (*p* = 0.63)	0.05 (*p* = 0.81)	0.00 (*p* = 0.98)	−0.27 (*p* = 0.17)
	Female	0.00 (*p* = 0.95)	−0.07 (*p* = 0.66)	0.11 (*p* = 0.52)	−0.0 (*p* = 0.57)	−0.26 (*p* = 0.12)

Controls	Male	−0.67 (*p* = 0.21)	0.67 (*p* = 0.21)	0.37 (*p* = 0.53)	0.31 (*p* = 0.60)	0.38 (*p* = 0.52)
	Female	−0.55 (*p* = 0.10)	−0.05 (*p* = 0.9)	0.25 (*p* = 0.48)	−0.05 (*p* = 0.87)	0.32 (*p* = 0.35)

**Table 6:** Correlation between abdominal lean mass and calcium hormones.

Group	Gender	Calcitonin	Osteocalcin	PTH	IGF-1	Vitamin D
Patients	Male	0.13 (*p* = 0.48)	0.30 (*p* = 0.12)	0.14 (*p* = 0.48)	0.12 (*p* = 0.53)	−0.14 (*p* = 0.48)
	Female	0.04 (*p* = 0.81)	0.01 (*p* = 0.94)	−0.30 (*p* = 0.15)	0.91 (*p* = 0.29)	0.83 (*p* = 0.25)

Controls	Male	−0.66 (*p* = 0.22)	0.34 (*p* = 0.57)	−0.30 (*p* = 0.62)	0.91 (*p* = 0.02)^†^	0.83 (*p* = 0.07)
	Female	−0.42 (*p* = 0.22)	−0.13 (*p* = 0.71)	0.39 (*p* = 0.26)	−0.03 (*p* = 0.91)	0.27 (*p* = 0.43)

^†^Statistically significant, *p* < 0.05.
